# A comparison of the outcomes of families with children aged less than 2 who received universal versus sustained nurse home visiting services in Korea: a cross-sectional study

**DOI:** 10.4178/epih.e2025004

**Published:** 2025-02-06

**Authors:** Yu-Mi Kim, Sun Hwa Park, Kyung Ja June, Sung-Hyun Cho, Ji Yun Lee, Hong-Jun Cho, Young-Ho Khang

**Affiliations:** 1 Department of Preventive Medicine, Hanyang University College of Medicine, Seoul, Korea; 2 Department of Nursing, Chung Cheong University, Cheongju, Korea; 3 Department of Nursing, Soonchunhyang University, Cheonan, Korea; 4 Institute of Health Policy and Management, Seoul National University Medical Research Center, Seoul, Korea; 5 College of Nursing, Research Institute of Nursing Science, Seoul National University, Seoul, Korea; 6 Department of Nursing, Kangwon National University, Chuncheon, Korea; 7 Department of Family Medicine, Asan Medical Center, University of Ulsan College of Medicine, Seoul, Korea; 8 Department of Health Policy and Management, Seoul National University College of Medicine, Seoul, Korea

**Keywords:** Maternal-child health service, Home visits, Nurse, Home environment, Cross-sectional study

## Abstract

**OBJECTIVES:**

This study aimed to compare maternal outcomes and the home environment between non‑vulnerable families with children under 2 receiving universal home visiting services and vulnerable families receiving sustained home visiting services.

**METHODS:**

This study was conducted in Seoul, Korea, where the country’s first nurse‑led early childhood home visiting program was introduced. A total of 551 mother‑child dyads participated in cross‑sectional surveys conducted at various child ages (6±2 weeks, 6±1 months, 12±1 months, and 24±1 months). Universal home visiting services were provided within six weeks postpartum to non‑vulnerable families, while vulnerable families received sustained services consisting of 25 visits over 24 months. Maternal knowledge of sudden infant death syndrome (SIDS) and childcare, maternal distress, and the Korean Infant‑Toddler Home Observation for Measurement of Environment (K‑IT‑HOME) were assessed.

**RESULTS:**

Overall, the universal home visitation group demonstrated higher levels of maternal knowledge regarding SIDS and childcare compared to the sustained home visitation group (all p-values <0.05), while the sustained home visitation group reported higher levels of maternal distress (p<0.001). The total K‑IT‑HOME score was 1.47 points higher in the universal home visitation group than in the sustained home visitation group (p<0.001). No significant differences were observed in the acceptance, organization, or involvement subscales of the K‑IT‑HOME (all p-values >0.05).

**CONCLUSIONS:**

This study demonstrated that disparities in maternal outcomes and home environments persisted in early childhood between the sustained and universal home visitation groups.

## GRAPHICAL ABSTRACT

[Fig f4-epih-47-e2025004]

## Key Message

This study is a cross-sectional survey comparing outcomes between universal home visitation group (non-vulnerable families) and sustained home visitation group (vulnerable families) at four time points: 6±2 weeks postpartum, 6±1 months postpartum, 12±1 months postpartum, and 24±1 months postpartum. Study results suggest that merely implementing a maternal and early childhood sustained home-visiting program is insufficient to close the gap in maternal outcomes and home environments.

## INTRODUCTION

Exposures to early life adversities—such as violence, household mental illness, or substance abuse—have long‑term harmful effects on children’s health and development [1]. Children from socioeconomically disadvantaged, ethnic minority, and indigenous backgrounds bear an unequal burden of these adversities [2]. Interventions provided during the prenatal and early childhood periods can significantly influence both childhood development and adult well‑being [3,4]. Targeted, intensive home visitation programs led by trained nurses from the prenatal period until the child reaches two years of age have been shown to positively affect outcomes for both children and parents, especially among disadvantaged and vulnerable families [5-10]. The “Closing the Gap in a Generation” report by the Commission on Social Determinants of Health highlighted that “equity from the start” is essential for achieving health and social equity later in life [11]. Home visiting programs for maternal and early childhood care, particularly those aimed at disadvantaged and vulnerable families, have been identified as a promising approach to narrow disparities in child health and development, thereby promoting health equity [5,11-14]. However, the effectiveness of targeted, intensive early childhood nurse home‑visiting interventions in bridging outcome gaps between vulnerable and non‑vulnerable families remains uncertain. Studies comparing outcomes between vulnerable families receiving intensive home‑visiting services and non‑vulnerable families not receiving such intensive services are sparse.

In Korea, the Seoul Healthy First Step Project (SHFSP) was launched in 2013 in the Seoul metropolitan area as a regional initiative to promote health equity [15]. The SHFSP employs a proportionate universality approach and consists of 2 main components: universal home visitation and selective sustained home visitation. The sustained component provides 25 home visits over the first 24 months postpartum (beginning prenatally) for high‑risk families facing socioeconomic disadvantages and psychosocial resource deficits. In contrast, the universal home visitation component typically involves a single visit within 6 weeks postpartum for families not identified as highly vulnerable during the initial risk screening [15]. As the SHFSP is a policy initiative aimed at reducing health inequalities, it raises an important research question: Are there differences in outcomes between the universal and sustained home visitation groups? This study compared maternal outcomes and the home environment among families with children under 2 receiving either universal or sustained home visiting services.

## MATERIALS AND METHODS

### Study design

This cross‑sectional survey examined whether differences in maternal outcomes and the home environment existed between the universal and sustained home visitation groups at 6±2 weeks, 6±1 months, 12±1 months, and 24±1 months postpartum. These time points correspond to changes in the sustained home visitation schedule (Supplementary Material 1) of the SHFSP, as well as the recommended deadline for universal home visits (within six weeks postpartum).

### Study setting

Seoul, the capital and largest city of Korea, is home to one‑fifth of the country’s population (51 million) and is divided into 25 administrative districts. In 2013, the Seoul Metropolitan Government initiated the SHFSP in three districts, gradually expanding the program to all 25 districts by 2020 [16]. Data collection occurred across 19 districts from January 2017 to November 2017. At the time of data collection in 2017, a total of 20 districts in Seoul were participating in the SHFSP.

### Universal and sustained home visitation

In Korea, national health insurance provides universal access to antenatal health services [17]. The average number of antenatal visits reached 18.7 in 2022 [17]. Other antenatal services—including iron and folic acid supplementation, provision of a maternal and child book, integrated tests for detecting chromosomal disorders, gestational diabetes testing, and maternal health checks—are publicly offered to all pregnant women through District Public Health Centers.

The universal home visitation program was established in 2013 as an integral component of the SHFSP, targeting families with newborns not identified as highly vulnerable during the initial screening. In 2017, the universal home visitation program covered 19.6% of all families with newborns in Seoul [16]. These visits typically occur within the first six weeks postpartum, with families receiving an average of 1.1 visits (standard deviation [SD], 0.3) [18]. During these visits, a range of topics is addressed, including the infant’s physical examination, assessment of growth and development, feeding practices, crying, child safety, and the mother’s physical and mental health. Each visit lasts approximately 80-85 minutes [16].

Sustained home visitation was provided to families identified as vulnerable during the initial screening. Vulnerability was assessed based on a combination of socio-demographic, health and healthcare, and psychosocial factors [15,16,18]. Socio-demographic factors included being a young mother (aged 23 or younger), having a low income (defined as families receiving basic living security or having a household income at or below 50% of the national median), coming from a multicultural background (born or raised abroad), having a disability, and being a single mother or part of a grandparent led family. Health and healthcare factors encompassed delayed prenatal care (initiation of obstetric care at 20 weeks of gestation or later) and substance use (such as smoking or alcohol consumption). Psychosocial factors, adapted from the SAFE START guidelines in New South Wales, Australia [19], included scores on the Edinburgh Postnatal Depression Scale (EPDS), lack of instrumental or emotional support, recent significant stressors, low self-esteem, feelings of anxiety or depression, a history of treatment for emotional issues, relationship problems or couple dysfunction, adverse childhood experiences, and support needs related to domestic violence. Families with 2 or more risk factors qualified for the sustained home visitation service. The Maternal and Early Childhood Sustained Home visitation (MECSH) program, developed in Australia, was implemented as the sustained home visitation service within the SHFSP [20,21]. The schedule for these visits is detailed in Supplementary Material 1. On average, each sustained home visit lasts approximately 60-65 minutes [16]. Although the standard schedule anticipates a minimum of 25 visits, a previous analysis showed that families who completed the sustained home visiting program received an average of 20.6 visits (SD, 5.6) by 24 months postpartum [18]. The content of each visit is tailored to the mother’s individual needs, skills, strengths, and capabilities, utilizing parenting education materials. Tailored interventions are provided to improve parenting skills and the home environment [15,16].

### Study participants

**Figure 1.**
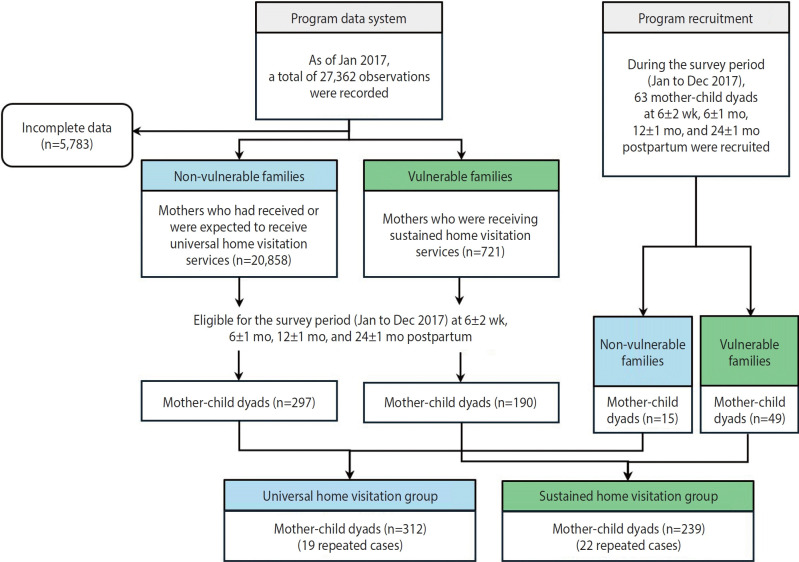
A flowchart on eligible population of the study and the study participants.

Eligibility criteria were applied for both the type of home visitation and the survey periods. First, eligible participants for the universal and sustained home visitation groups were identified using the SHFSP data system, which contains information on potential recipients of these services. Figure 1 presents a flowchart detailing the eligible population and study participants. According to the SHFSP data system, as of January 2017 there were 27,362 observations. After excluding 5,783 incomplete records, we identified 20,858 mothers who had received or were expected to receive universal home visitation services and 721 mothers receiving sustained home visitation services. We then selected eligible mother child dyads for the survey periods, whose children were at 6±2 weeks, 6±1 months, 12±1 months, and 24±1 months postpartum. Mother child dyads were contacted and recruited via telephone, resulting in the recruitment of 297 dyads from the universal program and 190 from the sustained program. Due to insufficient numbers of eligible dyads for certain survey periods (particularly at 24±1 months postpartum) in the sustained home visitation group, we waited for additional dyads (15 for non-vulnerable families and 49 for vulnerable families) to reach the survey periods. Four research nurses conducted home visits to collect data through face to face interviews and observations from January 2017 to November 2017. Final participants were mothers who consented for themselves, provided parental permission for their children, and voluntarily participated in the survey. During the survey periods, 239 mother child dyads in the sustained home visitation group participated (52 at 6±2 weeks, 77 at 6±1 months, 75 at 12±1 months, and 35 at 24±1 months postpartum), while 312 dyads participated in the universal home visitation group (74 at 6±2 weeks, 81 at 6±1 months, 78 at 12±1 months, and 79 at 24±1 months postpartum). Since the survey spanned 11 months, some participants (19 dyads in the universal home visitation group and 22 in the sustained home visitation group) were recruited twice.

### Compensation

Participants were compensated for their participation. Families in the universal home visitation program received 20,000 Korean won (with 1,300 Korean won equivalent to 1 US dollar), whereas families in the sustained home visitation program received 10,000 Korean won, acknowledging that they were already receiving ongoing home visitation services.

### Patient and public involvement

Patients and the general public were not involved in the design, conduct, or reporting of the study. The study’s objectives were disclosed to and approved by the participants (mothers) and the District Public Health Centers.

### Questionnaire and outcome measurements

To measure study outcomes, we recruited four research nurses (two for the first half of the survey period and two for the second half) and provided training in outcome measurement. The pre-trained research nurses conducted home visits to administer the survey questionnaires. Baseline characteristics collected included maternal age (in years), infant age (in months), number of children, monthly household income (in Korean won), parental education levels, and maternal depression status. Maternal depression was assessed using the 10‑item EPDS [22], with scores ranging from 0 to 30, where higher scores indicate more severe depressive symptoms.

Four outcome indicators were measured: maternal knowledge of sudden infant death syndrome (SIDS), maternal knowledge of childcare, maternal distress as measured by the 13‑item Being a Mother Scale (BaM‑13) [23], and the quality of the home environment as assessed by the Korean Infant‑Toddler Home Observation for Measurement of Environment (K‑IT‑HOME). The questionnaire on maternal knowledge of SIDS consists of 3 items addressing the supine position (placing infants on their backs during sleep), ensuring no head covering, and avoiding tobacco exposure, derived from the Korean National Health Insurance Service’s Infant Health Examination Questionnaire [24]. Maternal knowledge of SIDS was scored based on the number of correct responses, ranging from 0 to 3, with higher scores indicating greater knowledge of SIDS prevention. Maternal knowledge of childcare was evaluated using a 13‑item questionnaire from the Panel Study on Korean Children [25]. These items were adapted from the Knowledge of Infant Development Inventory originally developed by MacPhee [26]. The questionnaire assesses knowledge related to the cognitive, emotional, and social development of young children, with scores ranging from 0 to 13 based on the number of correct responses. The BaM‑13, developed by Matthey [23], comprises 13 items designed to assess maternal distress, with scores ranging from 0 to 39; higher scores indicate greater distress. The Korean version of the BaM‑13 was translated by the authors of this study and used to assess maternal distress in Korean women with children under 24 months [27]. The Infant‑Toddler Home Observation for Measurement of Environment (IT‑HOME) inventory assesses the quality and quantity of stimulation available to children in the home [28]. It includes 45 items across 6 subscales: responsivity, acceptance, organization, learning materials, involvement, and variety in daily stimulation. The Korean version of the inventory (K‑IT‑HOME) has been standardized for use with young Korean children [29]. Research nurses received practical training in administering the K‑IT‑HOME through structured interviews and family observations. Before measuring K‑IT‑HOME in study participants, we recruited 9 families (not included in the main study) for a reliability test (6 families for the first 2 research nurses and 3 families for the second 2). The K‑IT‑HOME instructor (serving as the gold standard) and the 4 trained research nurses conducted home visits and measured the K‑IT‑HOME for the same families. The reliability between the trained nurses’ assessments and the gold standard ranged from 89.8% to 96.0%.

### Statistical analysis

**Figure 2.**
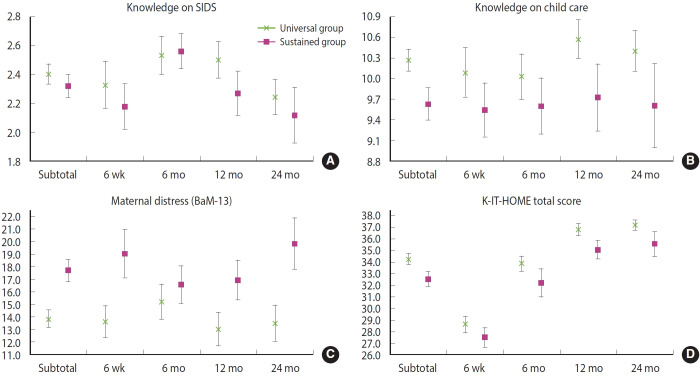
Comparison of scores (mean values and 95% confidence intervals) for maternal (A) knowledge of sudden infant death syndrome (SIDS) and (B) knowledge of child care, (C) maternal distress (Being a Mother 13 items, BaM-13), and (D) the home environment (Korean Infant-Toddler Home Observation for Measurement of Environment, K-IT-HOME) between the universal and sustained home visitation groups according to age of young children. For the comparison of subtotal scores involving four measurement periods, generalized linear mixed models accounting for random effects of repeatedly measured cases (19 cases in the universal home visitation group and 22 cases in the sustained home visitation group), and variance component covariance structure for random effects were used.

**Figure 3.**
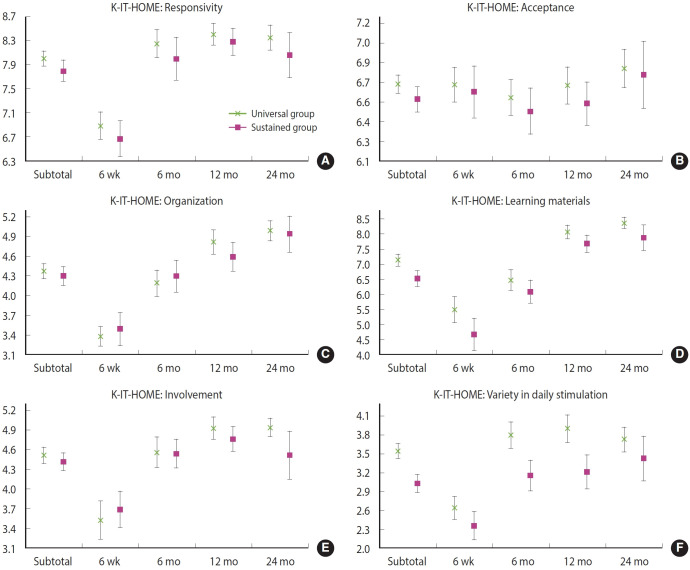
Comparison of scores (mean and 95% confidence intervals) for six subscales of Korean Infant-Toddler Home Observation for Measurement of Environment (K-IT-HOME) between the universal and sustained home visitation groups according to age of young children. The six subscales are: (A) Responsivity, (B) Acceptance, (C) Organization, (D) Learning materials, (E) Involvement, and (F) Variety in daily stimulation. For the comparison of subtotal scores involving four measurement periods, generalized linear mixed models accounting for random effects of repeatedly measured cases (19 cases in the universal home visitation group and 22 cases in the sustained home visitation group), and variance component covariance structure for random effects were used.

All analyses were performed using SAS version 9.4 (SAS Institute Inc., Cary, NC, USA), with a p‑value of less than 0.05 considered statistically significant. The characteristics of study participants were compared between the universal and sustained home visitation groups using the independent t‑test for continuous variables and the chi‑square test for categorical variables (Table 1). As the 4 outcome measures were continuous, their means at each measurement period were compared using the independent t‑test (Figures 2 and 3, Supplementary Materials 2 and 3). For comparisons of total scores across the 4 measurement periods, generalized linear mixed models were used to account for random effects from repeated measurements (19 cases in the universal home visitation group and 22 cases in the sustained home visitation group) with a variance component covariance structure (see total scores in Supplementary Materials 2 and 3). Three models were examined, each adjusting for different sets of covariates: (1) model 1 adjusted for maternal age and number of children, (2) model 2 included the model 1 covariates and household income and maternal education, and (3) model 3 included the model 2 covariates and EPDS. Maternal age and number of children were considered confounders in the relationship between family vulnerability (universal vs. sustained home visitation groups) and the outcome measures. Socioeconomic status indicators—household income and maternal education—and maternal depression (EPDS) could serve as mediators and/or confounders in the relationship between family vulnerability and the outcomes. Familial vulnerability (e.g., being a single mother or having a multicultural background) may lead to low socioeconomic status and maternal depression, which in turn could affect maternal knowledge, maternal distress, and the home environment. Alternatively, low socioeconomic status and maternal depression could determine familial vulnerability (since these factors are considered vulnerability factors) and also affect the outcome measures. We examined the extent to which the inclusion of socioeconomic status and maternal depression in the baseline model altered the absolute differences in the outcome measures.

### Ethics statement

All methods were conducted in accordance with relevant guidelines and regulations including the Declaration of Helsinki. The study protocol was approved by the Institutional Review Board of the Seoul National University Hospital (IRB No. C-1610-113-802). Participants (mothers) consented for themselves and gave parental permission for their babies. Participants were informed that their participation was voluntary, and their decision would not impact their care in any way.

## RESULTS

### Characteristics of study participants

Table 1 presents the characteristics of the participating mother‑child dyads. The 551 mothers had an average age of 32.6 years (SD, 5.1), and their babies had an average age of 10.4 months (SD, 8.0). More than two‑thirds of the families (68.6%) had one child, while only 5.4% had three or more children. Over half of the mothers (52.1%) and fathers (57.2%) had attained a university education or higher, whereas approximately a quarter of the mothers (25.2%) and a fifth of the fathers (21.8%) had completed high school or less. The mean EPDS score was 8.81 (SD, 5.90), with 40.7% of mothers scoring 10 or above.

Table 1 also shows differences between the universal and sustained home visitation groups. The average maternal age in the universal home visitation group was 33.4 years—1.8 years higher than the 31.6 years observed in the sustained home visitation group (p<0.001). The universal home visitation group was also socioeconomically better off; the average monthly household income was 1.72 million Korean won higher (5.23 vs. 3.51 million Korean won) than in the sustained home visitation group (p=0.001). Both mothers and fathers in the universal home visitation group were more highly educated than those in the sustained home visitation group (all p<0.001). Additionally, the sustained home visitation group had a higher EPDS score (p<0.001); 26.9% of mothers in the universal home visitation group had an EPDS score of 10 or higher, compared to 58.6% in the sustained home visitation group (p<0.001).

### Comparison of maternal outcomes and home environment

Figure 2 and Supplementary Material 2 present a comparison of maternal knowledge of SIDS and childcare, maternal distress, and the home environment between the universal and sustained home visitation groups, categorized by the child’s age.

Statistically significant differences were observed in the total scores for all four outcomes. Maternal knowledge scores for both SIDS and childcare were significantly higher in the universal home visitation group compared to the sustained home visitation group (p=0.020 and <0.001, respectively). Additionally, the total K‑IT‑HOME score was 1.47 points higher in the universal home visitation group than in the sustained home visitation group (p<0.001). Moreover, the total BaM‑13 score, indicating maternal distress, was lower in the universal home visitation group than in the sustained home visitation group (p<0.001).

However, some differences by time period were not statistically significant. At six months postpartum, there were no significant differences between the groups in maternal knowledge of SIDS, childcare, or BaM‑13 scores. Similarly, at six weeks postpartum the differences in maternal knowledge regarding both SIDS and childcare were not significant. In contrast, the differences in K‑IT‑ HOME scores remained significant across all four time periods.

Figure 2 also indicates that maternal knowledge of SIDS peaked at six months postpartum and that K‑IT‑HOME scores increased over time in both the universal and sustained home visitation groups.

### Comparison of subscales of the home environment

In contrast to the total K‑IT‑HOME score, the comparison between the universal and sustained home visitation groups showed variability across the six subscales (Figure 3, Supplementary Material 3). The scores for the acceptance, organization, and involvement subscales did not differ significantly between the two groups (p=0.089, 0.921, and 0.364, respectively), with the organization subscale scores being virtually identical. Across the four time periods, there were no significant differences between the groups in the responsivity, acceptance, or organization subscales. However, the involvement subscale showed a statistically significant difference at 24 months postpartum (p=0.041). In contrast, the subscales for learning materials and variety in daily stimulation generally exhibited significant differences across the time periods. Additionally, Figure 3 reveals that scores for all subscales, except for the acceptance subscale, increased over time in both groups.

### Comparison of covariate-adjusted outcomes

Table 2 presents a comparison of the covariate‑adjusted least square means (with standard errors) of outcome variables between the universal and sustained home visitation groups. The extent of outcome differences after adjustment varied by measure. The absolute differences between groups, adjusted in model 1 for maternal age and number of children, were similar to or slightly reduced compared with the unadjusted differences shown in Supplementary Material 2. For example, the unadjusted difference in the total K‑IT‑HOME score was 1.47, which decreased slightly to 1.36 in model 1. The difference in maternal knowledge of childcare scores decreased from 0.62 in model 1 to 0.44 in model 2, with no further reduction in model 3 (0.48). The difference in BaM‑13 scores was similar in models 1 (-3.98) and 2 (-3.93), but decreased significantly to -0.51 in model 3 after adjusting for maternal depression (EPDS), suggesting that the difference in maternal distress was largely explained by maternal depression. Table 2 also indicates that the difference in K‑IT‑HOME scores was partially explained by socioeconomic status and maternal depression. When household income and maternal education were included in model 2, the difference decreased from 1.36 to 1.11, and further to 0.84 in model 3 with the additional adjustment for EPDS.

## DISCUSSION

The results of this study present insights into the maternal outcomes and home environment for both the universal (non‑vulnerable families) and sustained (vulnerable families) home visitation groups over 24 months postpartum. Disparities between the two groups persisted throughout the study period despite the provision of selective and intensive home visiting services for high‑risk families by trained nurses. This suggests that bridging the gap in maternal outcomes and the home environment cannot be achieved solely through maternal and early childhood sustained home visitation programs. However, it is important to note the significant differences in monthly household income and parental education between the universal and sustained home visitation groups. The universal home visitation group had a 49% higher household income (5.23 vs. 3.51 million Korean won) and a lower proportion of mothers with only a high school education or less (15.7 vs. 37.7%) compared to the sustained home visitation group. To effectively close this gap, more comprehensive approaches addressing other social determinants over the life course—such as monetary and non‑monetary benefits for disadvantaged young women before pregnancy, enhanced antenatal healthcare, paid parental leave, and interventions addressing intimate partner violence—may be necessary [30-33].

Evidence suggests that gaps in maternal and childhood outcomes tend to widen as children age [34,35]. The literature indicates that early childhood disadvantages can serve as the origins of later health inequalities [36-38]. Vulnerable families (the sustained home visitation group) are more likely to experience adverse life events, making it plausible that the absolute gaps in maternal outcomes and the home environment observed in this study could have widened without sustained home visiting. In this context, the intensive maternal and early childhood home visitation service implemented here may have prevented further increases in these gaps, which typically occur as children grow older. A recent right@home trial based on the MECSH program (similar to the sustained home visitation program in this study) demonstrated statistically significant increases in the involvement and variety in stimulation subscales of the IT‑HOME inventory by 0.26 and 0.20, respectively [39]. In our study, the universal home visitation group exhibited an excess of 0.42 and 0.30, respectively, for these two IT‑HOME subscales at 24 months postpartum compared to the sustained home visitation group (Supplementary Material 3). If we hypothesize that sustained home visitation would have produced similar effects in the SHFSP, it could be suggested that about 40% of the total gap in these two IT‑HOME subscales—(0.26/[0.42+0.26]*100=38.2% for the involvement subscale; 0.20/[0.30+0.20]*100=40% for the variety in stimulation subscale)—between the universal and sustained home visitation groups were closed by the implementation of the sustained home visitation.

Despite the persistent gaps in the four main outcomes over time, no significant differences were observed in several K‑IT‑HOME subscales. Notably, gaps in the acceptance and organization subscales did not emerge. The acceptance subscale assesses behaviors such as physical punishment, shouting, slapping, scolding, or criticizing, while the organization subscale encompasses activities such as getting out of the house, taking a child to the grocery store, and maintaining the child’s play environment. These subscales are more closely related to maternal roles in parenting [28], suggesting that sustained home visitation may have helped narrow these specific gaps. However, significant differences were found in the learning materials and variety in stimulation subscales over the same period. The learning materials subscale assesses the availability of various toys, equipment, and learning facilitators, while the variety in stimulation subscale reflects the father’s role, family networks, and the child’s access to books. Given the differences in socioeconomic backgrounds between the universal and sustained home visitation groups, the persistent disparities in these subscales could not be readily addressed through sustained home visitation alone.

The study also indicated that there were no significant differences in three maternal outcomes at six months postpartum. Notably, maternal knowledge scores regarding SIDS showed a reversed pattern at this time point, possibly due to the increased frequency of visits prior to the 6‑month mark, as outlined in the sustained home visitation schedule (Supplementary Material 1).

This study demonstrated changes in the absolute differences of outcome measures when adjustments were made for low socioeconomic status and maternal depression. The disparity in BaM‑13 scores was accounted for by the EPDS. Socioeconomic status indicators along with EPDS partially explained the variance in K‑IT‑HOME scores. BaM‑13 assesses maternal distress [23], which is closely linked to maternal depression, while K‑IT‑HOME evaluates a broad spectrum of home environments conducive to child development. Certain items in K‑IT‑HOME—such as those in the learning materials and variety in stimulation subscales—may indicate family resources correlated with socioeconomic status, whereas items related to responsivity, acceptance, and involvement may reflect maternal availability associated with EPDS scores. These characteristics may explain the observed changes in outcome differences after adjusting for covariates.

This study has several limitations. First, as a cross‑sectional observational study, it provides only insights into the effect of the sustained home visitation intervention. To determine whether sustained home visiting has overcome inequitable outcomes between the universal and sustained home visitation groups, a stepped‑ wedge randomized controlled trial or a pre‑post examination of outcomes for both groups would be required. However, we lack baseline outcome data from before the implementation of sustained home visitation. Second, the study samples—particularly from the universal home visitation group—may not be representative of the target population. Eligible participants were initially selected from the SHFSP data system; as of January 2017, 721 families were enrolled in sustained home visitation (with a final sample size of 190), compared to 297 families recruited from non‑vulnerable families out of 20,858 registered mothers (Figure 1). Analyses of basic characteristics (maternal age, single motherhood status, disability status, and receipt of basic living security) did not reveal meaningful differences between non‑vulnerable mothers who were not surveyed and those who were. Third, the study did not provide evidence of the effects of sustained home visitation on outcomes; it only reported the outcome statuses of the universal and sustained home visitation groups. An ongoing randomized controlled community trial may offer more rigorous scientific evidence regarding the impact of sustained home visitation interventions in Korea [40].

In conclusion, the universal home visitation group exhibited higher maternal knowledge regarding SIDS and childcare, along with higher scores for the quality and quantity of stimulation available to children in the home, compared to the sustained home visitation group. In contrast, the sustained home visitation group experienced greater maternal distress. These findings indicate that the gap in maternal outcomes and home environments generally persisted in early childhood between the sustained and universal home visitation groups.

## Figures and Tables

**Figure f4-epih-47-e2025004:**
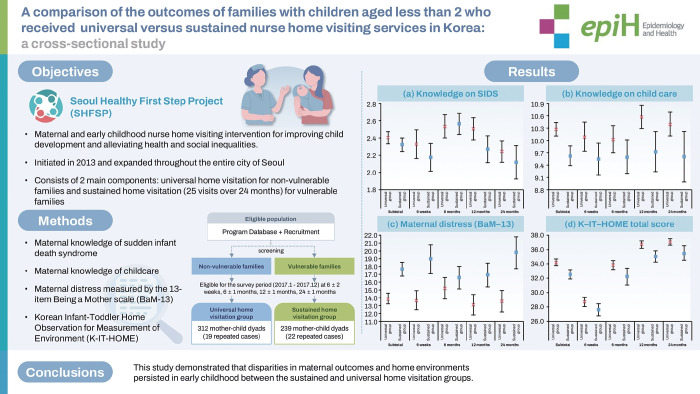


**Table 1.** Characteristics of study participants (mother-child dyads) who received universal or sustained home visitation services

Characteristics	Universal home visitation group	Sustained home visitation group	Total	p-value
Total no. of mother-child dyads	312	239	551	
Mother’s age (yr)	33.4±3.9	31.6±6.2	32.6±5.1	<0.001
Mean age of young children (mo)	11.2±8.5	9.4±7.1	10.4±8.0	0.009
6±2 wk	74 (23.7)	52 (21.8)	126 (22.9)	0.009
6±1 mo	81 (26.0)	77 (32.2)	158 (28.7)	
12±1 mo	78 (25.0)	75 (31.4)	153 (27.8)	
24±1 mo	79 (25.3)	35 (14.6)	114 (20.7)	
No. of children				0.032
1	225 (72.1)	153 (64.0)	378 (68.6)	
2	76 (24.4)	67 (28.0)	143 (26.0)	
3 or more	11 (3.5)	19 (8.0)	30 (5.4)	
Monthly household income (million Korean won)^1^	5.23±8.05	3.51±3.15	4.49±6.46	0.001
Mother’s education				<0.001
University or over	189 (60.6)	98 (41.0)	287 (52.1)	
Community college	73 (23.4)	51 (21.3)	124 (22.5)	
High school or less	49 (15.7)	90 (37.7)	139 (25.2)	
Missing	1 (0.3)	0	1 (0.2)	
Father’s education				<0.001
University or over	212 (68.0)	103 (43.1)	315 (57.2)	
Community college	50 (16.0)	44 (18.4)	94 (17.1)	
High school or less	48 (15.4)	72 (30.1)	120 (21.8)	
Missing	2 (0.6)	20 (8.4)	22 (4.0)	
EPDS	6.77±4.66	11.47±6.29	8.81±5.90	<0.001
EPDS≥10	84 (26.9)	140 (58.6)	224 (40.7)	<0.001

Values are presented as mean±standard deviation or number (%). SD, standard deviation; EPDS, Edinburgh Postnatal Depression Scale.

1 Missing=11.

**Table 2.** Comparison of covariate-adjusted least square mean (and SE) of outcome variables between the universal home visitation group and sustained home visitation group^1^

Variables	Universal home visitation group	Sustained home visitation group	Difference±SE	p-value
Model 1: Maternal age and no. of children adjusted				
Maternal knowledge of SIDS	2.45±0.05	2.29±0.05	0.16±0.06	0.007
Maternal knowledge of child care	10.03±0.14	9.40±0.14	0.62±0.15	<0.001
Maternal distress (BaM-13)	14.02±0.55	18.00±0.55	-3.98±0.59	<0.001
K-IT-HOME score	33.79±0.28	32.43±0.28	1.36±0.30	<0.001
Model 2: Model 1+Household income and maternal education adjusted				
Maternal knowledge of SIDS	2.45±0.05	2.31±0.05	0.14±0.06	0.025
Maternal knowledge of child care	9.94±0.14	9.51±0.14	0.44±0.15	0.007
Maternal distress (BaM-13)	14.10±0.57	18.03±0.57	-3.93±0.61	<0.001
K-IT-HOME score	33.67±0.29	32.56±0.29	1.11±0.31	0.001
Model 3: Model 2+EPDS adjusted				
Maternal knowledge of SIDS	2.45±0.05	2.31±0.06	0.14±0.06	0.034
Maternal knowledge of child care	9.96±0.14	9.48±0.15	0.48±0.16	0.006
Maternal distress (BaM-13)	15.19±0.42	15.70±0.43	-0.51±0.48	0.299
K-IT-HOME score	33.58±0.29	32.74±0.30	0.84±0.33	0.016

SE, standard error; SIDS, sudden infant death syndrome; BaM-13, Being a Mother 13 items; K-IT-HOME, Korean Infant-Toddler Home Observation for Measurement of Environment; EPDS, Edinburgh Postnatal Depression Scale.

1 Generalized linear mixed models accounting for random effects of repeatedly measured cases (19 cases in the universal home visitation group and 22 cases in the sustained home visitation group) and variance component covariance structure for random effects were used.

## Supplementary materials

Supplementary Material 1. Sustained home visitation schedule of the Seoul Healthy First Step Project

Supplementary Material 2. Comparison of maternal knowledge on sudden infant death syndrome (SIDS) and child care, maternal distress (Being a Mother 13 items), and home environment (K-IT-HOME) between universal versus sustained home visitation groups according to children's age

Supplementary Material 3. Comparison of six subsclales of Korean Infant-Toddler Home Observation for Measurement of Environment between universal and sustained home visitation groups according to children's age

## References

[1] Hughes K, Bellis MA, Hardcastle KA, Sethi D, Butchart A, Mikton C (2017). The effect of multiple adverse childhood experiences on health: a systematic review and meta-analysis. Lancet Public Health.

[2] O’Connor M, Slopen N, Becares L, Burgner D, Williams DR, Priest N (2020). Inequalities in the distribution of childhood adversity from birth to 11 years. Acad Pediatr.

[3] Campbell F, Conti G, Heckman JJ, Moon SH, Pinto R, Pungello E (2014). Early childhood investments substantially boost adult health. Science.

[4] Richter LM, Daelmans B, Lombardi J, Heymann J, Boo FL, Behrman JR (2017). Investing in the foundation of sustainable development: pathways to scale up for early childhood development. Lancet.

[5] Molloy C, Beatson R, Harrop C, Perini N, Goldfeld S (2021). Systematic review: effects of sustained nurse home visiting programs for disadvantaged mothers and children. J Adv Nurs.

[6] Olds DL, Kitzman H, Anson E, Smith JA, Knudtson MD, Miller T (2019). Prenatal and infancy nurse home visiting effects on mothers: 18-year follow-up of a randomized trial. Pediatrics.

[7] Kitzman H, Olds DL, Knudtson MD, Cole R, Anson E, Smith JA (2019). Prenatal and infancy nurse home visiting and 18-year outcomes of a randomized trial. Pediatrics.

[8] Conti G, Smith J, Anson E, Groth S, Knudtson M, Salvati A (2024). Early home visits and health outcomes in low-income mothers and offspring: 18-year follow-up of a randomized clinical trial. JAMA Netw Open.

[9] Goldfeld S, Bryson H, Mensah F, Price A, Gold L, Orsini F (2022). Nurse home visiting to improve child and maternal outcomes: 5-year follow-up of an Australian randomised controlled trial. PLoS One.

[10] Price A, Bryson H, Mensah FK, Kenny B, Wang X, Orsini F (2023). Embedding nurse home visiting in universal healthcare: 6-year follow-up of a randomised trial. Arch Dis Child.

[11] Marmot M, Friel S, Bell R, Houweling TA, Taylor S, Commission on Social Determinants of Health (2008). Closing the gap in a generation: health equity through action on the social determinants of health. Lancet.

[12] Duffee JH, Mendelsohn AL, Kuo AA, Legano LA, Earls MF, Council on Community Pediatrics (2017). Early childhood home visiting. Pediatrics.

[13] Finello KM, Terteryan A, Riewerts RJ (2016). Home visiting programs: what the primary care clinician should know. Curr Probl Pediatr Adolesc Health Care.

[14] https://www.instituteofhealthequity.org/resources-reports/fair-society-healthy-lives-the-marmot-review.

[15] Khang YH, Cho SH, June KJ, Lee JY, Kim YM, Cho HJ (2018). The Seoul Healthy First Step Project: introduction and expansion, program content and performance, and future challenges. J Korean Soc Matern Child Health.

[16] Khang YH, June KM, Cho SH, Lee JY, Kim YM, Cho HJ (2023). The 10th annual report of the Seoul Healthy First Step Project.

[17] Kim S, Kim C, Kim JH (2024). Antenatal care inequalities in South Korea: an analysis of health insurance claims data (2013-2022) in a high-resource, high-use country. Int J Gynaecol Obstet.

[18] Lee JY, Khang YH, June KJ, Cho SH, Cho HJ, Kim YM (2020). Final report on performance evaluation of Seoul Healthy First Step Project.

[19] https://www1.health.nsw.gov.au/pds/ActivePDSDocuments/GL2010_004.pdf.

[20] Kemp L, Harris E, McMahon C, Matthey S, Vimpani G, Anderson T (2011). Child and family outcomes of a long-term nurse home visitation programme: a randomized controlled trial. Arch Dis Child.

[21] Kemp L, Cowley S, Byrne F (2017). Maternal early childhood sustained home-visiting (MECSH): a UK update. J Health Visit.

[22] Cox J, Holden J, Henshaw C (2014). Perinatal mental health: the EPDS manual.

[23] Matthey S (2011). Assessing the experience of motherhood: the Being a Mother Scale (BaM-13). J Affect Disord.

[24] https://www.nhis.or.kr/nhis/healthin/retrieveInfntExmdDtInq.do.

[25] https://panel.kicce.re.kr/engpskc/board/index.do?menu_idx=41&amp;manage_idx=110.

[26] MacPhee D (2002). Knowledge of infant development inventory survey of child care experiences (KIDI): manual.

[27] Lee JY, Park SE, Kim YM, Cho HJ, Khang YH (2022). An analysis of the very high level of maternal distress experienced by South Korean women with young children. PLoS One.

[28] Caldwell BM, Bradley RH (2003). Home observation for measurement of the environment: administration manual.

[29] Lee Y, Lee JR, Park SJ, Woo HK, Koo JY, Chung HJ (2015). A study on the development of the normative scores for the IT-HOME inventory. Fam Environ Res.

[30] Phelan JC, Link BG, Tehranifar P (2010). Social conditions as fundamental causes of health inequalities: theory, evidence, and policy implications. J Health Soc Behav.

[31] Buultjens M, Farouque A, Karimi L, Whitby L, Milgrom J, Erbas B (2021). The contribution of group prenatal care to maternal psychological health outcomes: a systematic review. Women Birth.

[32] Heshmati A, Honkaniemi H, Juárez SP (2023). The effect of parental leave on parents’ mental health: a systematic review. Lancet Public Health.

[33] Bacchus LJ, Colombini M, Pearson I, Gevers A, Stöckl H, Guedes AC (2024). Interventions that prevent or respond to intimate partner violence against women and violence against children: a systematic review. Lancet Public Health.

[34] Bahk J, Yun SC, Kim YM, Khang YH (2015). Changes in the relationship between socioeconomic position and maternal depressive symptoms: results from the panel study on Korean children (PSKC). Matern Child Health J.

[35] Beeghly M, Olson KL, Weinberg MK, Pierre SC, Downey N, Tronick EZ (2003). Prevalence, stability, and socio-demographic correlates of depressive symptoms in Black mothers during the first 18 months postpartum. Matern Child Health J.

[36] Shonkoff JP (2012). Leveraging the biology of adversity to address the roots of disparities in health and development. Proc Natl Acad Sci U S A.

[37] Melchior M (2021). Invited commentary: is the long shadow of childhood disadvantage on lifelong health getting worse over time?. Am J Epidemiol.

[38] Woolfenden S, Goldfeld S, Raman S, Eapen V, Kemp L, Williams K (2013). Inequity in child health: the importance of early childhood development. J Paediatr Child Health.

[39] Goldfeld S, Price A, Smith C, Bruce T, Bryson H, Mensah F (2019). Nurse home visiting for families experiencing adversity: a randomized trial. Pediatrics.

[40] Khang YH, Kim YM, Kim JH, Yu J, Oh R, June KJ (2024). Impact of the Korea Early Childhood Home-visiting Intervention (KECHI) on child health and development and maternal health: a randomised controlled trial protocol. BMJ Open.

