# Supplementary material for: A comparison of the outcomes of families with children aged less than 2 who received universal versus sustained nurse home visiting services in Korea: a cross-sectional study
Source: Epidemiol Health. 2025 Feb 6;47:e2025004. doi: 10.4178/epih.e2025004 (PMC12062853; doi:10.4178/epih.e2025004)
Supplement: Supplementary Material 1. — Sustained home visitation schedule of the Seoul Healthy First Step Project [file epih-47-e2025004-Supplementary-1.docx]

Supplementary Material 1. Sustained home visitation schedule of the Seoul Healthy First Step Project

| Child age (weeks) | Minimum no. of visits | Frequency of visits |
| --- | --- | --- |
| Before birth | 3 |  |
| 0-6 weeks | 5 | Weekly |
| 7-12 weeks | 3 | Fortnightly |
| 13-26 weeks | 4 | 3-weekly |
| 27-52 weeks | 4 | 6-weekly |
| 53-104 weeks | 6 | Bimonthly |
