# Supplementary material for: A comparison of the outcomes of families with children aged less than 2 who received universal versus sustained nurse home visiting services in Korea: a cross-sectional study
Source: Epidemiol Health. 2025 Feb 6;47:e2025004. doi: 10.4178/epih.e2025004 (PMC12062853; doi:10.4178/epih.e2025004)
Supplement: Supplementary Material 2. — Comparison of maternal knowledge on sudden infant death syndrome (SIDS) and child care, maternal distress (Being a Mother 13 items), and home environment (K-IT-HOME) between universal versus sustained home visitation groups according to children's age [file epih-47-e2025004-Supplementary-2.docx]

Supplementary Material 2. Comparison of maternal knowledge on sudden infant death syndrome (SIDS) and child care, maternal distress (Being a Mother 13 items), and home environment (K-IT-HOME) between universal versus sustained home visitation groups according to children's age

|  | N | Total |  | N | Universal home visitation group |  | N | Sustained home visitation group | Difference  ±SE | P value |
| --- | --- | --- | --- | --- | --- | --- | --- | --- | --- | --- |
|  | N | Mean±SE |  | N | Mean±SE |  | N | Mean±SE |  |  |
| Maternal knowledge on SIDS | | |  |  |  |  |  |  |  |  |
| 6±2 weeks | 126 | 2.26±0.67 |  | 74 | 2.32±0.72 |  | 52 | 2.17±0.58 | 0.15±0.12 | 0.215 |
| 6±1 months | 158 | 2.54±0.58 |  | 81 | 2.53±0.61 |  | 77 | 2.56±0.55 | -0.03±0.09 | 0.767 |
| 12±1 months | 153 | 2.39±0.64 |  | 78 | 2.50±0.58 |  | 75 | 2.27±0.68 | 0.23±0.10 | 0.024 |
| 24±1 months | 114 | 2.20±0.57 |  | 79 | 2.24±0.56 |  | 35 | 2.11±0.58 | 0.13±0.12 | 0.275 |
| Total | 551 | 2.36±0.63 |  | 312 | 2.40±0.63 |  | 239 | 2.32±0.63 | 0.13±0.06 | 0.020 |
| Maternal knowledge on child care | | |  |  |  |  |  |  |  |  |
| 6±2 weeks | 126 | 9.86±1.54 |  | 74 | 10.08±1.59 |  | 52 | 9.54±1.43 | 0.54±0.28 | 0.052 |
| 6±1 months | 158 | 9.82±1.68 |  | 81 | 10.02±1.51 |  | 77 | 9.60±1.82 | 0.43±0.27 | 0.110 |
| 12±1 months | 153 | 10.15±1.81 |  | 78 | 10.56±1.28 |  | 75 | 9.72±2.15 | 0.84±0.29 | 0.004 |
| 24±1 months | 114 | 10.15±1.54 |  | 79 | 10.39±1.32 |  | 35 | 9.60±1.85 | 0.79±0.35 | 0.026 |
| Total | 551 | 9.99±1.66 |  | 312 | 10.27±1.44 |  | 239 | 9.62±1.85 | 0.66±0.15 | <.0001 |
| Maternal distress (Being a Mother 13 items) | | | | | |  |  |  |  |  |
| 6±2 weeks | 126 | 15.84±6.66 |  | 74 | 13.61±5.40 |  | 52 | 19.02±7.03 | -5.41±1.16 | <.0001 |
| 6±1 months | 158 | 15.86±6.58 |  | 81 | 15.19±6.49 |  | 77 | 16.57±6.65 | -1.39±1.05 | 0.187 |
| 12±1 months | 153 | 14.93±6.73 |  | 78 | 13.01±5.96 |  | 75 | 16.92±6.95 | -3.91±1.04 | 0.0003 |
| 24±1 months | 114 | 15.44±7.06 |  | 79 | 13.49±6.57 |  | 35 | 19.83±6.19 | -6.33±1.31 | <.0001 |
| Total | 551 | 15.51±6.74 |  | 312 | 13.84±6.16 |  | 239 | 17.69±6.84 | -4.03±0.58 | <.0001 |
| K-IT-HOME score | |  |  |  |  |  |  |  |  |  |
| 6±2 weeks | 126 | 28.13±3.00 |  | 74 | 28.59±2.87 |  | 52 | 27.49±3.08 | 1.10±0.55 | 0.046 |
| 6±1 months | 158 | 33.07±4.20 |  | 81 | 33.85±2.85 |  | 77 | 32.21±5.19 | 1.64±0.69 | 0.019 |
| 12±1 months | 153 | 35.95±3.00 |  | 78 | 36.78±2.30 |  | 75 | 35.06±3.41 | 1.72±0.48 | 0.001 |
| 24±1 months | 114 | 36.69±2.45 |  | 79 | 37.17±1.97 |  | 35 | 35.58±3.07 | 1.59±0.58 | 0.009 |
| Total | 551 | 33.51±4.61 |  | 312 | 34.24±4.18 |  | 239 | 32.53±4.96 | 1.47±0.30 | <.0001 |

SIDS: Sudden infant death syndrome, SE: Standard error, SD: Standard deviation, K-IT-HOME: Korean Infant-Toddler Home Observation for Measurement of Environment

For the comparison of total scores involving four measurement periods, generalized linear mixed models accounting for random effects of repeatedly measured cases (19 cases in the universal home visitation group and 22 cases in the sustained home visitation group), and variance component covariance structure for random effects were used.
