# Supplementary material for: A comparison of the outcomes of families with children aged less than 2 who received universal versus sustained nurse home visiting services in Korea: a cross-sectional study
Source: Epidemiol Health. 2025 Feb 6;47:e2025004. doi: 10.4178/epih.e2025004 (PMC12062853; doi:10.4178/epih.e2025004)
Supplement: Supplementary Material 3. — Comparison of six subsclales of Korean Infant-Toddler Home Observation for Measurement of Environment between universal and sustained home visitation groups according to children's age [file epih-47-e2025004-Supplementary-3.docx]

Supplementary Material 3. Comparison of six subsclales of Korean Infant-Toddler Home Observation for Measurement of Environment between universal and sustained home visitation groups according to children's age

|  | N | Total |  | N | Universal home visitation group |  | N | Sustained home visitation group | Difference  ±SE | P value |
| --- | --- | --- | --- | --- | --- | --- | --- | --- | --- | --- |
|  | N | Mean±SE |  | N | Mean±SE |  | N | Mean±SE |  |  |
| Responsivity |  |  |  |  |  |  |  |  |  |  |
| 6±2 weeks | 120 | 6.79±1.01 |  | 69 | 6.88±0.95 |  | 51 | 6.67±1.09 | 0.22±0.19 | 0.246 |
| 6±1 months | 151 | 8.13±1.31 |  | 79 | 8.25±1.03 |  | 72 | 8.00±1.57 | 0.25±0.22 | 0.248 |
| 12±1 months | 148 | 8.34±0.89 |  | 77 | 8.40±0.82 |  | 71 | 8.28±0.97 | 0.12±0.15 | 0.413 |
| 24±1 months | 110 | 8.26±1.00 |  | 77 | 8.35±0.96 |  | 33 | 8.06±1.09 | 0.29±0.21 | 0.165 |
| Total | 529 | 7.91±1.24 |  | 302 | 8.00±1.12 |  | 227 | 7.80±1.37 | 0.22±0.10 | 0.032 |
| Acceptance |  |  |  |  |  |  |  |  |  |  |
| 6±2 weeks | 120 | 6.66±0.63 |  | 69 | 6.68±0.56 |  | 51 | 6.63±0.72 | 0.05±0.12 | 0.658 |
| 6±1 months | 150 | 6.53±0.68 |  | 79 | 6.58±0.61 |  | 71 | 6.48±0.75 | 0.10±0.11 | 0.356 |
| 12±1 months | 148 | 6.61±0.68 |  | 77 | 6.68±0.64 |  | 71 | 6.54±0.71 | 0.14±0.11 | 0.209 |
| 24±1 months | 110 | 6.79±0.68 |  | 77 | 6.81±0.65 |  | 33 | 6.76±0.75 | 0.05±0.14 | 0.738 |
| Total | 528 | 6.64±0.67 |  | 302 | 6.69±0.62 |  | 226 | 6.57±0.73 | 0.11±0.06 | 0.089 |
| Organization |  |  |  |  |  |  |  |  |  |  |
| 6±2 weeks | 120 | 3.43±0.75 |  | 69 | 3.38±0.62 |  | 51 | 3.49±0.90 | -0.11±0.15 | 0.442 |
| 6±1 months | 150 | 4.24±0.97 |  | 79 | 4.19±0.92 |  | 71 | 4.30±1.03 | -0.11±0.16 | 0.508 |
| 12±1 months | 148 | 4.71±0.88 |  | 77 | 4.82±0.82 |  | 71 | 4.59±0.93 | 0.23±0.14 | 0.119 |
| 24±1 months | 110 | 4.97±0.71 |  | 77 | 4.99±0.68 |  | 33 | 4.94±0.79 | 0.05±0.15 | 0.749 |
| Total | 528 | 4.34±1.02 |  | 302 | 4.37±0.99 |  | 226 | 4.30±1.05 | 0.01±0.08 | 0.921 |
| Learning materials | |  |  |  |  |  |  |  |  |  |
| 6±2 weeks | 120 | 5.14±1.92 |  | 69 | 5.49±1.84 |  | 51 | 4.67±1.95 | 0.83±0.35 | 0.019 |
| 6±1 months | 150 | 6.29±1.60 |  | 79 | 6.47±1.57 |  | 71 | 6.08±1.63 | 0.38±0.26 | 0.144 |
| 12±1 months | 148 | 7.88±1.14 |  | 77 | 8.06±1.02 |  | 71 | 7.68±1.24 | 0.39±0.19 | 0.038 |
| 24±1 months | 110 | 8.22±0.99 |  | 77 | 8.36±0.83 |  | 33 | 7.88±1.24 | 0.48±0.24 | 0.046 |
| Total | 528 | 6.88±1.89 |  | 302 | 7.14±1.78 |  | 226 | 6.53±1.97 | 0.47±0.13 | 0.001 |
| Involvement |  |  |  |  |  |  |  |  |  |  |
| 6±2 weeks | 120 | 3.59±1.13 |  | 69 | 3.52±1.22 |  | 51 | 3.69±1.01 | -0.16±0.21 | 0.434 |
| 6±1 months | 150 | 4.55±1.00 |  | 79 | 4.56±1.06 |  | 71 | 4.54±0.94 | 0.02±0.16 | 0.895 |
| 12±1 months | 148 | 4.84±0.78 |  | 77 | 4.92±0.76 |  | 71 | 4.76±0.80 | 0.16±0.13 | 0.209 |
| 24±1 months | 110 | 4.81±0.81 |  | 77 | 4.94±0.64 |  | 33 | 4.52±1.06 | 0.42±0.20 | 0.041 |
| Total | 528 | 4.47±1.06 |  | 302 | 4.51±1.09 |  | 226 | 4.41±1.01 | 0.08±0.09 | 0.364 |
| Variety in daily stimulation | | |  |  |  |  |  |  |  |  |
| 6±2 weeks | 120 | 2.52±0.80 |  | 74 | 2.64±0.77 |  | 51 | 2.35±0.82 | 0.28±0.15 | 0.053 |
| 6±1 months | 150 | 3.49±1.04 |  | 81 | 3.80±0.95 |  | 71 | 3.15±1.04 | 0.64±0.16 | 0.0001 |
| 12±1 months | 148 | 3.57±1.13 |  | 78 | 3.90±0.98 |  | 71 | 3.21±1.17 | 0.68±0.18 | 0.0002 |
| 24±1 months | 110 | 3.64±0.94 |  | 79 | 3.73±0.88 |  | 33 | 3.42±1.03 | 0.30±0.19 | 0.120 |
| Total | 528 | 3.32±1.09 |  | 312 | 3.54±1.03 |  | 226 | 3.03±1.10 | 0.50±0.09 | <.0001 |

SE: Standard error, K-IT-HOME: Korean Infant-Toddler Home Observation for Measurement of Environment

For the comparison of total scores involving four measurement periods, generalized linear mixed models accounting for random effects of repeatedly measured cases (19 cases in the universal home visitation group and 22 cases in the sustained home visitation group), and variance component covariance structure for random effects were used.
